# Treatment of Chronic Migraine with Focus on Botulinum Neurotoxins

**DOI:** 10.3390/toxins7072615

**Published:** 2015-07-14

**Authors:** Sara M. Schaefer, Christopher H. Gottschalk, Bahman Jabbari

**Affiliations:** Department of Neurology, Yale University, 20 York St., New Haven, CT 06510, USA; E-Mails: Chris.Gottschalk@yale.edu (C.H.G.); Bahman.Jabbari@yale.edu (B.J.)

**Keywords:** botulinum toxin, migraine, headache, chronic migraine, chronic daily headache, neurotoxins, medication overuse headache

## Abstract

Migraine is the most common neurological disorder, and contributes to disability and large healthcare costs in the United States and the world. The treatment of migraine until recently has focused on medications, both abortive and prophylactic, but treatment of chronic migraine has been revolutionized with the introduction of botulinum toxin injection therapy. In this review, we explore the current understanding of migraine pathophysiology, and the evolution of the use of botulinum toxin therapy including proposed pathophysiological mechanisms through animal data. We also discuss the similarities and differences between three injection techniques.

## 1. Introduction

Migraine is the most common neurological disorder, and the third most prevalent condition overall in the world, behind anemia and hearing loss [1]. It has a characteristic throbbing quality, is of moderate to severe intensity, is generally unilateral, and has associated symptoms including photophobia, phonophobia, and gastrointestinal distress, though all of these may not be present in each patient [2]. Those with a severe migraine attack are often described as seeking a quiet, dark room because routine activities exacerbate the headache. Migraine may occur with or without aura, which may be visual, sensory, or dysphasic, is described as episodic (occurring less than 15 days per month) or chronic (occurring greater than or equal to 15 days per month), and may occur with or without medication overuse.

Migraine affects 18% of women and 6% of men, with the highest prevalence among those aged 30–39 (24.4% of women and 7.4% of men) [3]. The direct and indirect annual costs were reported as high as $17 billion dollars in the United States in 2005 [4] and more recently 27 billion Euro/year in Italy [5].

## 2. Pathophysiology

The aura phase of migraine likely corresponds to the phenomenon of cortical spreading depression (CSD), an electrical event demonstrated in animal models that travels across the cortex at a speed of 3–6 mm per second and often involves the occipital cortex, leading to visual aura [6]. Release of potassium, nitric oxide, adenosine, and other agents during CSD may cause inflammation and vasodilation in the cortex and meningeal vessels [7]. The sensitized trigeminovascular system sends enhanced afferent impulses to the trigeminal ganglion, trigeminal nucleus caudalis, superior salivatory nucleus, and parasympathetic efferent fibers [8]. One downstream effect of this neurochemical cascade is dural vasodilation, with calcitonin gene-related peptide (CGRP) representing one of many key players in the process [9]. The resultant nociceptive stimuli at different levels of the nervous system and at the trigeminal nuclei evoke head and facial pain. Recent studies have demonstrated a potential role of transient receptor potential vanilloid type-1 receptor (TRPV1) and transient receptor potential ankyrin 1 (TRPA1) channels in the pathophysiology of migraine pain. Del Fiacco *et al.* demonstrated an increase of TRPV1 in periarterial nerve fibers from scalp artery specimens in migraine patients as compared to controls, irrespective of whether or not the patient had a migraine at the time of sampling, implying a more chronic uptick in TRPV1 receptors in these patients [10]. Many common triggers of migraine act as TRPA1 agonists, thereby inducing the release of CGRP [9]. Cutaneous allodynia during a migraine attack may mark a transition of pain from peripheral to central, given that peripherally activating agents such as triptans are ineffective once patients reach a stage of allodynia [11]. Migraine has been described as a “vascular headache”; however, the vasodilation and increased cerebral blood flow previously cited as potential causes of migraine pain are not temporally correlated with headache—headache pain starts before vasodilation [12]. Therefore, vasodilation may exacerbate rather than originate pain. Moreover, the throbbing nature of migraine pain does not correspond to a patient’s pulse when measured simultaneously [13].

A recent review in genetics of migraine demonstrated that the first-degree relatives of migraine patients with and without aura have 4 and 1.9 times the risk of developing the same type of migraine, respectively [14]. Co-occurrence was noted in 52% of female twins raised together or apart. Cernuda-Morollon *et al.* found that serum levels of calcitonin gene-related peptide (CGRP) are 2.5 times higher in patients with chronic migraine (CM) compared to asymptomatic controls and about 1.8 times higher in patients with episodic migraine (EM) or cluster headaches (*p* < 0.05), identifying a potential biomarker for primary headache disorders [15].

## 3. Treatment

Treatment of migraine consists of abortive and preventive therapy [16,17]. Acetaminophen, aspirin, and non-steroidal anti-inflammatory drugs (NSAIDs) are often used for management of mild attacks. For more severe attacks triptans are recommended. However, many patients with migraine respond poorly to triptans, and there are contraindications such as cardiovascular co-morbidities that make triptans difficult to prescribe [18]. Intravenous administration of some combination of dopamine receptor agonists (e.g., prochlorperazine), dihydroergotamine (DHE), and intravenous (IV) NSAIDs (diclofenac or ketorolac) is recommended for more severe episodes, especially those attacks that have surpassed the peripheral phase of activation [19]. High oxygen flow may alleviate acute attacks of migraine [20].

Preventive daily treatment of migraine is recommended when migraine episodes exceed 6–8 headache days per month or what is tolerable to the patient, if the patient has to use abortive medications more than 8–9 times per month, or if headache-related disability is significant [21]. Beta-blockers such as propranolol or metoprolol, topiramate, amitriptyline, and divalproex sodium (DVPX) are commonly used for migraine prevention [22].

## 4. Botulinum Toxins (BoNTs): Animal Data

Emerging animal data indicate that BoNTs can influence pain, peripheral sensitization, and central sensitization through a number of different mechanisms.

### 4.1. Peripheral Effects

In addition to its known selective inhibition of acetylcholine release from cholinergic nerve endings at the skeletal neuromuscular junction, BoNT type A has peripheral effects on pain and inflammation by directly influencing neurotransmitter release at sensory nerve endings. A recent study by Burstein *et al.* found that BoNT-A administration directly to C-meningeal nociceptors in the dura inhibited responses to mechanical stimulation, reversed mechanical hypersensitivity, and prevented development of mechanical hypersensitivity [23]. BoNT type A inhibits the release of pain peptides, substance P, bradykinin, CGRP, and glutamate *in vivo* from the dorsal root and trigeminal ganglia [24,25,26]. Shimizu *et al.* showed that BoNT-A injection into the ophthalmic division of the trigeminal ganglion decreased TRPV1 immunoreactive neurons by decreasing TRPV1 trafficking to the plasma membrane, an effect that persisted for at least 14 days [27]. In the rat bladder, BoNT type A inhibits both cholinergic and purinergic inputs by inhibiting release of acetylcholine and adenosine triphosphate from muscle efferents, leading to reduction of painful bladder spasms [28]. In the formalin model of pain, injection of BoNT type A into the rat paw a week prior to formalin injection reduces the post-formalin inflammatory peak of pain in a dose-related manner. Tissue examination of the injected site reveals decreased inflammation and decreased local glutamate accumulation compared to controls [29]. BoNT inhibits a family of G proteins, including Rho Guanosine triphosphatase, which are essential for activation of interleukin-1, an important pro-inflammatory cytokine [30]. Intra-prostatic injection of BoNT type A inhibits cyclooxygenase-2 expression and suppresses capsaicin-induced prostatitis in the animal model [31]. Luvisetto *et al.* have postulated that in the capsaicin model of pain, the reduction in pain with BoNT pretreatment may be due to its downregulation of TRPV1 responsiveness to capsaicin, a TRPV1 agonist [32]. Femtomolar concentrations of BoNT type A inhibit membrane sodium channels in rat central and peripheral neurons [33]; over-activity of these channels contributes to chronic and neuropathic pain. Local onabotulinum toxin A (ona-A) injection impairs sympathetic transmission [34], thus interfering with maintenance of pain by decreasing sympathetic overactivity. Intramuscular injection of ona-A decreases the discharge of muscle spindles, a major sensory input to the spinal cord [35].

### 4.2. Central Effects

Recently the focus of antinociceptive effects of BoNT has shifted to central effects, particularly through sensory and motor nerve retrograde axonal transport. Studies demonstrate bilateral effects of BoNT following unilateral injection of the toxin indicating potential central etiology [36]. Further studies demonstrate detection of BoNT type A truncated SNAP-25 in the dorsal horn of the trigeminal nucleus caudalis following peripheral injection of BoNT type A into the rat whisker pad [37], in the ipsilateral dorsal and ventral horns of the spinal cord following toxin injection into the sciatic nerve [38,39], and in spinal astrocytes following peripheral sciatic nerve injection [39]. Notably, simultaneous administration of colchicine (which inhibits retrograde axonal transport) negates any antinociceptive effect as observed by rat behavior, highlighting the importance of axonal transport on the effects of BoNT type A.

## 5. Botulinum Toxin Treatment of Chronic Migraine

Chronic migraine is defined as a headache with a frequency of 15 or more headache days per month (at least 8 migraine type), for more than three months, lasting more than 4 hours per day [2]. The pioneering study of BoNT in episodic migraine had some positive results [40], which generated interest in the study of BoNT treatment efficacy in all forms of migraine. However, results of subsequent studies of BoNT in episodic migraine were negative, leading to the designation of level A evidence for ineffectiveness in episodic migraine from the Therapeutics and Technology Assessment Subcommittee of the American Academy of Neurology 2008 [41]. Early studies of BoNT’s effect on chronic daily headache (including a large number of subjects with chronic migraine) also produced contradictory results, casting a shadow of doubt on the efficacy of ona-A for chronic migraine. However, Dodick *et al.* looked at the efficacy of ona-A in 228 patients with no prophylactic medications and found a statistically significant difference in relief compared to placebo at successive time points over three months (*p* = 0.004, *p* = 0.032 and *p* = 0.023), which opened the door to further investigation [42].

Freitag *et al.*, in a double-blind placebo-controlled study, compared the effect of a fixed-dose (100 units), fixed-site (glabella, frontalis, temporal, trapezius, suboccipital) paradigm treatment with ona-A (20 patients) *vs.* placebo (21 patients) in chronic migraine [43]. All patients with medication overuse were excluded. The primary outcome was the number of migraine episodes. The secondary outcomes were number of headache days and headache index (HI, a measure of both intensity and frequency). Ona-A was statistically superior to placebo for both primary (*p* < 0.01) and secondary outcomes (frequency of pain days *p* = 0.041 at 4 weeks and *p* = 0.046 at 16 weeks, and HI *p* = 0.003 at 16 weeks).

In the summer of 2010, the results of Phase III Research Evaluating Migraine Prophylaxis Therapy (PREEMPT) 1 and PREEMPT 2 trials, two class I multicenter studies assessing efficacy of ona-A in chronic migraine, were published [44,45]. Each study included approximately 700 patients, with comparable numbers of subjects in the toxin and placebo groups, in a 24-week blind arm followed by a 32-week open arm. Both studies included patients with medication overuse. The primary outcome for PREEMPT 1 was the number of headache episodes, and for PREEMPT 2 the number of headache days, evaluated at 24 weeks. A number of secondary outcomes were evaluated at the 24-week time point, including frequency of moderate/severe headache days and cumulative headache hours. Although PREEMPT 1 did not meet the primary outcome, it met its secondary outcomes. PREEMPT 2 met its primary outcome with a decrease in headache days by 9 in the ona-A *vs.* 6.7 in the placebo groups (*p* < 0.001). The pooled data from the two studies also showed a significant change from baseline in favor of ona-A regarding the primary and secondary outcome parameters [46]. The FDA considered headache days (as in PREEMPT 2) a better outcome measure than headache episodes (as in PREEMPT 1) for the study of chronic migraine. Ona-A was approved for the treatment of chronic migraine in the UK, Canada, and the US in 2010.

Given the link between migraine pain and TRPs as discussed above, perivascular injections rather than classic muscular injections are being explored in animal models, and considered in humans [32,47].

## 6. Comparator Studies of BoNTs *vs.* Oral Agents in Chronic Migraine

### 6.1. Botulinum Toxin vs. Divalproex Sodium

In a single-center, double-blind, randomized prospective trial, Blumenfeld *et al.* explored the efficacy and tolerability of BoNT *vs.* DVPX in patients with episodic or chronic migraine [48]. Fifty-nine patients received either BoNT plus oral placebo or placebo injections plus DVPX 250 mg BID. Injections were given at the start and at month 3, with evaluations occurring at intervals to nine months. Outcome measures included reduction in number of headache days, responder rate (percentage of patients with a ≥50% reduction in attack frequency per month), maximum headache severity, and overall headache index (related to combination of headache frequency and severity). Patients in both groups demonstrated significant improvements in migraine frequency and severity as measured by headache days per month, responder rates, Headache Index scores, Migraine Disability Assessment (MIDAS), and Headache Impact Test (HIT-6) scores. A greater percentage of DVPX patients reported adverse events (AEs) (DVPX 75.8% *vs.* BoNTA 50%, *p* = 0.04), and those patient were more likely to discontinue treatment because of AEs (DVPX 27.6% *vs.* BoNTA 3.3%, *p* = 0.012). Therefore, while these treatment strategies are similarly efficacious, the side effect profile for BoNT was more favorable than for DVPX.

### 6.2. Botulinum Toxin vs. Topiramate

In a similar study, Mathew and Jaffri compared the relative efficacies, tolerability, and safety of botulinum toxin and topiramate in patients with CM without medication overuse [49]. Subjects were randomized into two groups: BoNT plus oral placebo and topiramate plus injected placebo. As in Blumenfeld’s study above, subjects were injected at time points 0 and 3 months and followed for a total of nine months. The primary endpoint was the Physician Global Assessment; the secondary endpoints included number of headache days, HIT-6, and MIDAS scores. The ona-A and topiramate groups demonstrated similar efficacies, with 40.9% and 42.9% respectively noting at least a 50% reduction in headache days after nine months. The marked improvement rates in headaches—at least 75% improvement on the Physician Global Assessment—at 9 months differed (27.3% for ona-A *vs.* 60.9% for topiramate, *p* = 0.0234); however, ona-A injections were only given at the beginning of the study and at month 3 while topiramate therapy was continued throughout the 9-month study. The remainder of data for the primary and secondary endpoints demonstrated similar outcomes between groups. Although nearly all study participants reported at least one adverse effect, the topiramate group permanently discontinued treatment due to AEs at a rate of 24.1%, compared to 7.7% in the ona-A group.

Another study evaluated BoNT *vs.* topiramate in CM patients using a 3-month protocol of treatment plus placebo [50]. The primary endpoint was Physician Global Assessment scores as above; the secondary endpoints included headache days, HIT-6, and MIDAS scores. No significant difference in efficacy was discovered between BoNT and topiramate, confirming the findings of Mathew and Jaffri in 2009 [49]. AEs were not assessed.

Earlier reports of PREEMPT results and that of comparator studies of ona-A proved short-term efficacy and safety in chronic migraine. However, patients and clinicians questioned log-term efficacy, safety, and changes in quality of life after BoNT treatment. Furthermore, since approximately two thirds of the patients in the PREEMPT studies had medication overuse, the efficacy of BoNTs in migraine patients with *vs.* without medication overuse needed clarification. Two recent studies convincingly answered these issues. 

## 7. The Issue of Medication Overuse in Chronic Migraine

Silberstein *et al.* studied the efficacy of ona-A in a subgroup of PREEMPT study patients who had both medication overuse and chronic migraine (MO+CM) [51]. Of the 1384 patients in the two PREEMPT studies, 65.3% met criteria for medication overuse. At 24 weeks, MO+CM patients demonstrated significant reduction of headache days compared to placebo (−8.2 *vs.* −6.2, *p* < 0.001) and also met many secondary endpoints (frequency of migraine days, frequency of moderate to severe headache days, cumulative headache hours on headache days, headache episodes, migraine episodes, and percentage of patients with severe Headache Impact Test-6 scores (all *p* < 0.05)). Triptan intake was significantly reduced in MO+CM patients after ona-A treatment (*p* < 0.001). The authors concluded that ona-A treatment is effective in patients with MO+CM in addition to those without medication overuse.

## 8. Long-Term Efficacy, Safety, and Effects on Quality of Life

Aurora *et al.* studied the efficacy of botulinum toxin and changes in quality of life in PREEMPT patients after five cycles of treatment (at 56 weeks) [52]. The mean decreases in headache days, migraine days, and moderate to severe headache days were all significantly improved (*p* < 0.05) compared to placebo. Quality of life improved by 44% at week 25 and 59% at week 56 (measured by a >5 point increase in HIT-6 scores). These findings therefore demonstrate increasing efficacy of ona-A in migraine with repeated treatments, as well as progressive improvement in quality of life over time. In a more recent study Silberstein *et al.* reported on the percent of patients with chronic migraine who responded to the onabotulinumA toxin treatment cycle in the PREEMPT studies [51]. Of 688 patients who received onaA, 49.3% described more than a 50% improvement after the first cycle of treatment, and an additional 11.3% and 10.3% after the second and third cycles of treatment. These data further support that repeat injections in subsequent cycles increases the efficacy of onaA in chronic migraine.

## 9. Techniques of Injection

**Figure 1:**
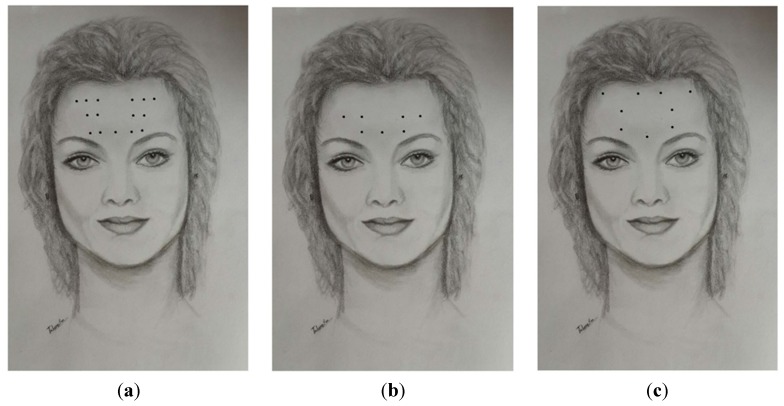
A comparison of the procerus, corrugator, and frontalis injection sites by Blumenfeld *et al.* 2003 (**a**); Blumenfeld *et al.* 2010 *i.e.*, PREEMPT (**b**); and Jabbari (Yale) 2015 (**c**).

**Figure 2:**
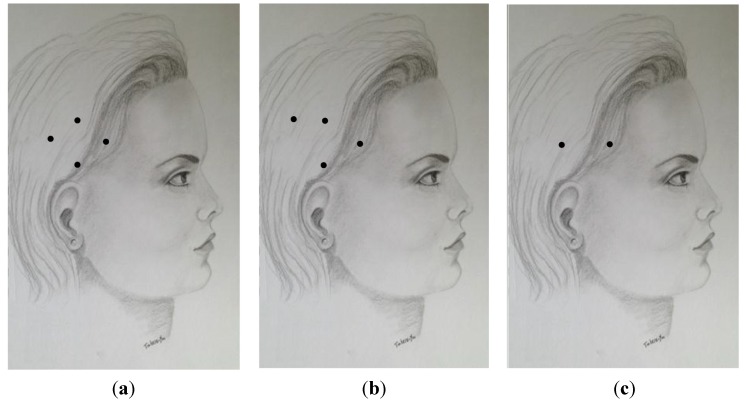
A comparison of the temporalis injection sites by Blumenfeld *et al.* 2003 (**a**); Blumenfeld *et al.* 2010 *i.e.*, PREEMPT (**b**); and Jabbari (Yale) 2015 (**c**).

**Figure 3:**
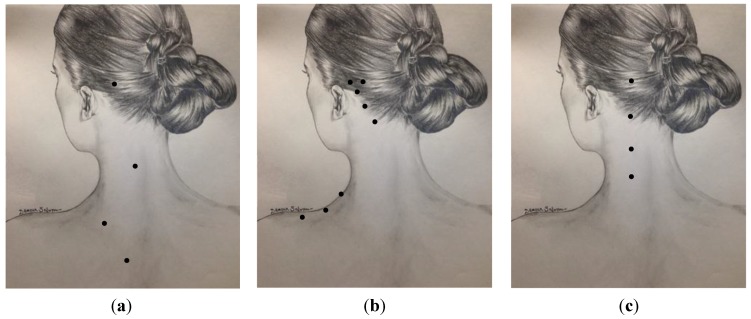
A comparison of the occipitalis, splenius/paraspinalis, and trapezius injection sites by Blumenfeld *et al.* 2003 (**a**); Blumenfeld *et al.* 2010 *i.e.*, PREEMPT (**b**); and Jabbari (Yale) 2015 (**c**).

A variety of techniques have been used for the treatment of CM with BoNT injections. All advocate procerus, corrugator, frontalis, temporalis, occipitalis and posterior cervical injections. In one of the earliest publications and widely used injection schemes [53], authors advocated for several frontal injections (5 sites per frontalis) with small doses of 2.5 units per site in addition to two injections into each corrugator and one into the procerus muscle at midline (Table 1, Figure 1a, Figure 2a and Figure 3a). A slight modification of this technique was used by Silberstein at Jefferson’s Headache Center until 2009 [54]. These methods consisted of 32 injection sites for a total dose of 130–160 units. The PREEMPT study recommends two injections of five units each into the frontalis, three into the occipitalis and three into the trapezius muscle on each side (Table 1, Figure 1b, Figure 2b and Figure 3b) [55]. PREEMPT uses a total of 31 injection sites and 165–195 total units. Table 1, Figure 1c, Figure 2c and Figure 3c demonstrate a technique used by Jabbari and some of his colleagues at Yale for the past 12 years [56]. In this technique each frontalis muscle is injected at three points with five units. The technique emphasizes the role of temporalis and posterior neck muscles in migraine which are injected with higher doses than those of the PREEMPT study. Trapezius muscles are not included in the plan of injection. It has the advantage of using fewer injection sites (23 *vs.* 31 in the PREEMPT studies), and uses a total dose of 185–195 units. We have prospectively recorded the response of 50 patients with CM who were treated with this technique (with onaA) and with repeated injections. Patient satisfaction was assessed by patient global impression of change (PGIC). Seventy two percent of the patients reported the level of change as “much improved” after the first injection and 85% reached the same level of satisfaction after several cycles of treatment (follow-up 2–8 years). Fifty percent of patients discontinued preventive medication and 61% of patients discontinued abortive medication by 12 months of treatment. Of the 15 patients who had been to the emergency department for headache within one year of starting treatment, 73% had no visits to the emergency department over the course of a year after receiving 12 months of treatment. No significant side effects were reported. Currently, ona-A (Botox- Allergan Inc. of Dublin, Ireland and Parsippany-Troy Hills, NJ, USA) is the only type of botulinum toxin approved by the FDA for the treatment of chronic migraine.

**Table 1:** Comparison of three techniques of ona-A treatment in CM: Muscles, doses in units, and number of injections per side.

Method	Procerus (Midline)	Corrugator	Frontalis	Temporalis	Occipitalis	Splenius/Paraspinalis	Trapezius	Masseter
Blumenfeld *et al.* 2003 [53]; Silberstein 2009 [54] (Figure 1a, Figure 2a and Figure 3a)	2.5–5 u	2.5 u × 2	2.5 u × 5	2.5–5 u × 4	2.5–5 u × 1	2.5–5 u × 1	2.5 u × 2	2.5–5 u × 1
PREEMPT Blumenfeld 2010 [55] (Figure 1b, Figure 2b and Figure 3b)	5 u	5 u × 1	5 u × 2	5 u × 4	5 u × 3	5 u × 2	5 u × 3	-
Jabbari (Yale) 2015 [56], (Figure 1c, Figure 2c and Figure 3c) *	5 u	5 u × 1	5 u × 3	15 u × 2	10 u × 1	10 u × 3	-	-

* There are other injectors at Yale who use the PREEMPT method for treatment of migraine.

## 10. Conclusions

Ona-A is now an established and major treatment mode for chronic migraine. Patient satisfaction with initial treatment is high and it increases with subsequent treatments. BoNT treatment of CM is safe and, over time, improves quality of life significantly. Different techniques of injection are available with positive and comparable results.
